# The synergistic effect of cigarette taxes on the consumption of cigarettes, alcohol and betel nuts

**DOI:** 10.1186/1471-2458-7-121

**Published:** 2007-06-25

**Authors:** Jie-Min Lee

**Affiliations:** 1 Department of Logistics Management, National Kaohsiung Marine University, N0.142, Hai-Chuan Rd. Nan-Tzu, Kaohsiung, Taiwan

## Abstract

**Background:**

Consumption of cigarettes and alcoholic beverages creates serious health consequences for individuals and overwhelming financial burdens for governments around the world. In Asia, a third stimulant – betel nuts – increases this burden exponentially. For example, individuals who simultaneously smoke, chew betel nuts and drink alcohol are approximately 123 times more likely to develop oral, pharyngeal and laryngeal cancer than are those who do not.

To discourage consumption of cigarettes, the government of Taiwan has imposed three taxes over the last two decades. It now wishes to lower consumption of betel nuts. To assist in this effort, our study poses two questions: 1) Will the imposition of an NT$10 Health Tax on cigarettes effectively reduce cigarette consumption? and 2) Will this cigarette tax also reduce consumption of alcoholic beverages and betel nuts? To answer these questions, we analyze the effect of the NT$10 tax on overall cigarette consumption as well as the cross price elasticities of cigarettes, betel nuts, and alcoholic beverages.

**Methods:**

To establish the Central Bureau of Statistics demand function, we used cigarette, betel nut, and alcoholic beverage price and sales volume data for the years 1972–2002. To estimate the overall demand price elasticity of cigarettes, betel nuts, and alcoholic beverages, we used a seemingly unrelated regression analysis.

**Results:**

We find that the NT$10 health tax on cigarettes will reduce cigarette consumption by a significant 27.22%. We also find that cigarettes, betel nuts, and alcoholic beverages have similar inherent price elasticities of -0.6571, -0.5871, and -0.6261 respectively. Because of this complementary relationship, the NT$10 health tax on cigarettes will reduce betel nut consumption by 20.07% and alcohol consumption by 7.5%.

**Conclusion:**

The assessment of a health tax on cigarettes as a smoking control policy tool yields a win-win outcome for both government and consumers because it not only reduces cigarette consumption, but it also reduces betel nut and alcoholic beverage consumption due to a synergistic relationship. Revenues generated by the tax can be used to fund city and county smoking control programs as well as to meet the health insurance system's current financial shortfall.

## Background

In Taiwan, as in countries around the world, cigarettes and alcohol pose many health threats. For instance, habitual smoking and excessive drinking can cause cardiovascular disease, digestive tract disease, hypertension and diabetes; they also increase the incidence of, and mortality from, many types of cancer [1-3]. Furthermore, studies have shown that alcohol abuse commonly leads to driving while intoxicated as well as to violent behavior [4,5].

In addition to the well-known, detrimental effects of alcohol and cigarettes, a third stimulant – betel nuts – also poses a serious health threat in Taiwan. Betel nut use has long flourished throughout Asia [6-9]. In fact, it is estimated that roughly 10%-20% of the world's population uses it [10,11]. This means that betel nut consumption ranks fourth (behind cigarettes, alcoholic beverages, and coffee) on the list of the world's most widely used central nervous system stimulants [12,13]. In 2002, Taiwan's Bureau of Health Promotion took a nationwide survey and found that 17% of men and 1.2% of women habitually chew it [14].

Betel nut use is closely correlated with oral diseases, such as mouth cancer and fibrosis of the oral mucosa [15]. In Taiwan, deaths from oral cancer increased four-fold (from 349 to 1439) in the 15-year period between 1986 and 2001, and the age-adjusted mortality rate more than doubled from 4.56 per 100,000 to 11.42 in the same time period. Oral cancer has now become the fifth-most prevalent cancer among Taiwanese males [16]. As a result of these and similar experiences around the world, the international medical community now believes that betel nuts are carcinogenic [17].

Individuals who simultaneously smoke, chew betel nuts, and drink alcohol are approximately 123 times more likely to develop oral, pharyngeal, and laryngeal cancer than are those who do not [15]. Unsurprisingly, many of the world's countries now consider control of the use and abuse of tobacco, alcohol, and betel nut as three of their major health priorities.

### Cigarette consumption

The price of cigarettes in Taiwan is much lower than in many other countries, which has long made it difficult to significantly reduce the smoking population. If we take the average amount of work needed to purchase a pack of cigarettes as a proxy for cigarette purchasing power, then it would take an Indian 77 minutes of work, an Indonesian 62 minutes, a Chinese 56 minutes, and a Taiwanese only 7–10 minutes to purchase a pack of domestic cigarettes. In 2002, the country had more than 4.5 million smokers, which implies that one out of every three adults is a smoker [18].

Apart from the direct toll that smoking takes in sickness and loss of life, it is estimated that health problems derived from it add approximately NT$20 billion annually to Taiwan's health insurance costs. It is also estimated that smoking reduced the GDP by more than US$1.032 billion in 2000[19].

Raising taxes on cigarettes is currently one of the most effective and widely-used policies with which governments around the world control tobacco use [20-24]. The World Health Organization (WHO) has also proposed the use of taxes and other control measures to lower the consumption of tobacco [25]. The government of Taiwan took the first steps to adopt such a taxation policy in 1987 when it imposed an in-kind tax on cigarettes that increased the price by NT$16.6 per pack [26]. After joining the World Trade Organization (WTO) in 2002, Taiwan imposed the *Wine and Tobacco Tax Act*, which added an excise tax of NT$11.8 per pack. The act also included an additional Health & Welfare tax of NT$5 per pack.

Prior to this act, the price of cigarettes in Taiwan had remained constant for many years. Following its implementation, however, the price of domestic cigarettes rose by close to 30%, and the price of a pack of cigarettes rose by roughly NT$10 to an average of approximately NT$45. This jump in cigarette prices caused consumption to fall from 2.31 billion packs in 2001 to 1.735 billion packs in 2002 [27]. The prevalence of smoking likewise fell – from 24.74% in 2001 to 23.53% in 2002; it fell again in 2003 to 22.84%. This indicates that cigarette price increases do indeed reduce both cigarette consumption and the prevalence of smoking [28]. In 2006, the government increased the health tax on cigarettes from NT$5 to NT$10.

### Alcohol and betel nut taxes

In addition to levying taxes on tobacco, Taiwan also taxes alcoholic beverages. Each liter of beer is subject to a tax of NT$26, and each liter of distilled liquor is subject to a tax of NT$185. Depending on alcohol content, reprocessed alcoholic beverages are taxed NT$7 per each percent of alcohol when the alcohol content is less than 20% by volume and NT$185 per liter when the alcohol content is over 20%.

It should be noted that no health tax has yet been imposed on either alcohol or betel nuts. Furthermore, no excise tax has yet been imposed on betel nuts.

### Consumption trends of both betel nuts and alcoholic beverages

Prior to 1987, consumption of betel nuts was negligible. However, between 1987 and 2001, the average retail price of betel nuts fell approximately 13.35% due to an oversupply. As a result, domestic consumption rose by 108%, and average annual consumption in 2001 increased to 9.3 kg per person. The number of betel nut users likewise rose significantly. For example, in the southern counties of Taiwan, 9.8% of all adults and 22.5% of smokers, respectively, consumed betel nuts prior to 1987. By 2001, the rates had risen to 17.0% and 31.3% [29].

Consumption patterns of alcoholic beverages have also changed over the last decade. The average retail price of alcoholic beverages fell during the period of 1996–2001, while the consumption of alcoholic beverages remained flat, with average consumption staying at approximately 43 liters per person in 2001. As the price of alcoholic beverages fell, the prevalence of drinking rose. Among citizens over 15 years of age, 34% of men and 23% of women drank alcoholic beverages in 2002 [30,31].

### The goals of this study

Over the last two decades, the government of Taiwan has attempted to discourage the consumption of cigarettes by imposing a series of taxes; in addition, discouraging consumption of betel nuts has now become one of its major health goals [32]. To assist in this effort, we began this study by posing two questions: 1) Will the imposition of an NT$10 health tax on cigarettes effectively reduce cigarette consumption; and 2) Will this tax on cigarettes also lower consumption of alcoholic beverages and betel nuts?

Our questions are based on the premise that smoking, drinking, and chewing betel nuts are different types of consumer behavior; in physiological terms, alcohol and betel nuts are not as addictive as the nicotine found in tobacco. Yet the three habits are linked together because many people who consume one product also consume the others. Specifically, this study assessed the effect of the NT$10 health tax imposed on each pack of cigarettes in 2006 on cigarette, betel nut, and alcoholic beverage consumption using the inherent price elasticity and the cross price elasticities of cigarettes-betel nuts and cigarettes-alcoholic beverages. The study also sought to assess the cross effect of the health tax on consumption of betel nuts and alcoholic beverages.

The results of this study indicate that the NT$10 health tax will not only reduce cigarette consumption, but it will also reduce betel nut and alcohol consumption. Such results provide an important reference with which to guide future decisions concerning health taxes imposed on cigarettes.

### An overview of cross price elasticity

Because of the possible linkage between cigarettes, betel nuts, and alcoholic beverages in consumer decision-making, this study performed the simultaneous estimation of the price elasticity of all three products. Because it used the information contained in the residuals, this approach avoids estimation bias and yields better results than single equations do.

The chief basis for assessing the effect of cigarette taxes on cigarette consumption and on consumption of betel nuts and alcoholic beverages is the inherent price elasticity of cigarettes and the cross price elasticity of cigarette price elasticity relative to betel nuts and of cigarettes relative to alcoholic beverages. Past studies of the inherent price elasticity of cigarettes mostly obtained price elasticity values in the range of -0.5~-1.0 for cigarettes in mid-/low-income countries and -0.25~-0.5 in high-income countries [33]. Most literature on the price elasticity of cigarettes in Taiwan gives figures in the range of -0.5~-1.1. This indicates that cigarette price hikes are more effective at reducing consumption in Taiwan than in higher income countries [34,35].

Literature on the cross price elasticity of cigarettes suggests that the cross price elasticities of cigarettes-alcoholic beverages and of cigarettes-betel nuts may be either negative or positive. Friend and Pagano (2005) showed that smokers whose cigarette consumption decreased were significantly less likely to revert to alcohol use than were those whose consumption increased or remained unchanged [36]. Most foreign estimates of the cross price elasticities of cigarettes-alcoholic beverages and cigarettes-betel nuts are in the range of -0.5~1.0 [37-41].

## Methods

### Study design

Price elasticities for cigarettes, betel nuts, and alcohol can be obtained statistically using a demand model. Demand for these products in Taiwan can be described using the Central Bureau of Statistics (CBS) (Keller and Van Driel, 1985) demand model [42], which, Lee *et al*. (2005) found to outperform other demand models in a comparative study [35]. The CBS demand model describes differential change in quantitative share as a function of changes in total cigarette, betel nut, and alcohol expenditures and prices:

wi,t(dlog⁡qi,t−dlog⁡Qt)=cidlog⁡Qt+∑jsi,jdlog⁡pj,t
 MathType@MTEF@5@5@+=feaafiart1ev1aaatCvAUfKttLearuWrP9MDH5MBPbIqV92AaeXatLxBI9gBaebbnrfifHhDYfgasaacH8akY=wiFfYdH8Gipec8Eeeu0xXdbba9frFj0=OqFfea0dXdd9vqai=hGuQ8kuc9pgc9s8qqaq=dirpe0xb9q8qiLsFr0=vr0=vr0dc8meaabaqaciaacaGaaeqabaqabeGadaaakeaacqWG3bWDdaWgaaWcbaGaemyAaKMaeiilaWIaemiDaqhabeaakiabcIcaOiabdsgaKjGbcYgaSjabc+gaVjabcEgaNjabdghaXnaaBaaaleaacqWGPbqAcqGGSaalcqWG0baDaeqaaOGaeyOeI0IaemizaqMagiiBaWMaei4Ba8Maei4zaCMaemyuae1aaSbaaSqaaiabdsha0bqabaGccqGGPaqkcqGH9aqpcqWGJbWydaWgaaWcbaGaemyAaKgabeaakiabdsgaKjGbcYgaSjabc+gaVjabcEgaNjabdgfarnaaBaaaleaacqWG0baDaeqaaOGaey4kaSYaaabuaeaacqWGZbWCdaWgaaWcbaGaemyAaKMaeiilaWIaemOAaOgabeaakiabdsgaKbWcbaGaemOAaOgabeqdcqGHris5aOGagiiBaWMaei4Ba8Maei4zaCMaemiCaa3aaSbaaSqaaiabdQgaQjabcYcaSiabdsha0bqabaaaaa@6823@

Where *q*_*i,t *_and *p*_*j,t *_are the quantity and the price of the *i*^*th *^good during period *t*, *w*_*i,t *_is the budget share of the *i*^*th *^good during period *t*, and dlog⁡Qt=∑iwi,tdlog⁡qi,t
 MathType@MTEF@5@5@+=feaafiart1ev1aaatCvAUfKttLearuWrP9MDH5MBPbIqV92AaeXatLxBI9gBaebbnrfifHhDYfgasaacH8akY=wiFfYdH8Gipec8Eeeu0xXdbba9frFj0=OqFfea0dXdd9vqai=hGuQ8kuc9pgc9s8qqaq=dirpe0xb9q8qiLsFr0=vr0=vr0dc8meaabaqaciaacaGaaeqabaqabeGadaaakeaacqWGKbazcyGGSbaBcqGGVbWBcqGGNbWzcqWGrbqudaWgaaWcbaGaemiDaqhabeaakiabg2da9maaqafabaGaem4DaC3aaSbaaSqaaiabdMgaPjabcYcaSiabdsha0bqabaGccqWGKbazcyGGSbaBcqGGVbWBcqGGNbWzcqWGXbqCdaWgaaWcbaGaemyAaKMaeiilaWIaemiDaqhabeaaaeaacqWGPbqAaeqaniabggHiLdaaaa@4971@ is the Divisia quantity index. The parameter *c*_*i *_denotes marginal budget share, while the parameter *s*_*ij *_measures the cross substitution effects of a compensated change in the price of *j *on the quantity of *i*. The theory of demand implies the following adding-up, symmetry, and homogeneity restrictions on the CBS parameters in equation (1). Finally, the Marshallian own-price and expenditure elasticities, respectively *e*_*ii *_and *η*_*i*_, can be derived from equation (1) as follows: *e*_*ii *_= (*s*_*ii*_/w_*i*_+c_*i*_)-*w*_*i *_and *ηi *= (*c*_*i*_/*w*_*i*_)+1. We calculated potential reductions in cigarette, betel nut, and alcohol consumption after levying the NT$10 tobacco health tax by multiplying price elasticity by the percentage of the price change. In this study, the CBS model constrained by homogeneity and symmetry conditions was implemented with Zellner's Seemingly Unrelated Regression (SUR) procedure using the Time Series Processor package (TSP), version 4.2B.

### Data

We used aggregate time series data for the period from 1972 through 2002 to analyze consumer demand for cigarettes, betel nuts, and alcohol. Data on cigarette and alcohol sales in Taiwan were obtained from the Taiwan Tobacco and Wine Monopoly Bureau (1972–2000) and National Treasury Agency (2001–2002) [43,27]. Because we could not obtain the real sales data for betel nuts, we have had to use production data of betel nuts for the period of 1972–2002, chiefly obtained from the ROC (Republic of China) Agricultural Statistics Yearbook, to calculate betel nut consumption [44]. It should be noted that the import of betel nuts is currently prohibited in Taiwan and that most betel nuts produced in the country are for domestic consumption, not export.

Cigarette, betel nut and alcohol consumption was calculated as the annual per capita number of packs of cigarettes, betel nuts, and alcoholic beverages sold to users 15 years of age or older. Annual per capita cigarette, betel nut and alcohol consumption by users 15 years of age or older was calculated by dividing total annual consumption by the year-end population of adults 15 years of age or older. (Note that cigarettes include both domestic and imported brands.) The total expenditures were classified as those on domestic cigarettes, imported cigarettes, betel nuts, wine, beer, and spirits. The year-end data for adults 15 years of age or older used in this study were obtained from the Ministry of the Interior's 2003 Statistical Yearbook of Interior [45].

Cigarette retail price data from 1972 to 2002 consisted of the average retail price per pack weighted by the quantity of each brand of cigarettes sold in Taiwan. Cigarette retail price data from 2001 to 2002 was obtained from the Taiwan Interview Survey on Cigarette Consumption (TISCC) (Tsai, 2005) [28]. The farm price of betel nuts was calculated as the betel nut production value divided by betel nut production quantity. All betel nut retail prices were derived by adding sales margin to betel farm prices. They are based on data from the Taiwan Agricultural Prices and Costs Monthly and the Taiwan Statistical Yearbook [46]. The price of alcohol was derived by dividing the total sales income of alcohol by total sales quantity of consumed alcohol. All cigarette, betel nut, and alcohol prices were deflated using the Consumer Price Index (1996).

## Results

### Elasticity of cigarette demand

Table 1 shows that estimated own price elasticity coefficients for cigarettes, alcohol, and betel nuts are -0.6517, -0.5871 and -0.6261 respectively. All of the own price elasticities (diagonal elements in Table 1) are negative and are statistically significant at a five percent level. All goods have own price elasticities greater than 0.5. This is a clear indication that consumption of cigarettes, alcohol, and betel nuts in Taiwan does respond to a change in price, although proportionate increases in price lead to a slightly less than proportionate reduction in consumption.

**Table 1:** Estimated price and expenditure elasticity values^a^

Item	Budget share	Expenditure	Prices		
			
			Cigarettes	Alcohol	Betel nuts
		1.0062	**-0.6517**	*-0.2503*	*-0.1041*
Cigarettes	0.439				
		(4.708)**	(-6475)**	(-2.842)**	(-2.526)**
		0.8104	*-0.1881*	**-0.5871**	*-0.0351*
Alcohol	0.401				
		(2.433)**	(-1.247)	(-4.407)**	(-0.546)
		1.4578	*-0.4840*	*-0.3476*	**-0.6261**
Betel nuts	0.160				
		(1.116)	(-0.857)	(-0.857)	(-2.481)**

Notes: *t *ratios are shown in parentheses.

a. Marshallian own-price elasticities

** Statistically significant at 5% level

Analysis of cross-price elasticities suggests the nature of the relationship between two products and indicates whether they are mutually substitutive or complementary. It implies, for instance, that betel nuts and cigarettes are complementary if cross-price elasticities for betel nuts are negative. Taking betel nuts as an example, the respective cross-price elasticity of -0.484 for cigarettes implies that demand for betel nuts is more sensitive to changes in the price of cigarettes. This figure implies that a 10% increase in the price of cigarettes will cause a 4.84% decrease in the price of betel nuts. Taking cigarettes as an example, demand for cigarettes is sensitive to changes in the price of alcohol (-0.2503), but less sensitive to changes in the price of betel nuts (-0.1041). This suggests that a change in the price of alcoholic beverages may cause more than double the change in the consumption of cigarettes when compared to the same change in the price of betel nuts.

### Effects of price changes due to taxation

This study used cigarette, betel nut, and alcoholic beverage consumption information for 2001 as baseline data to analyze the effect of two health tax increases on cigarette consumption, as well as the effect of the NT$10 health tax imposed on each pack of cigarettes in 2006, on the consumption of alcoholic beverages and betel nuts. The results of the calculations are shown in Table 2. Note that in 2001, average annual cigarette consumption in Taiwan was 126.35 packs per capita, and the average retail price was approximately NT$36 per pack. The price of cigarettes rose NT$15 (approximately 41.47%) after imposition of the NT$10 Health Tax in 2006.

**Table 2:** Effects of health taxes on cigarette (betel nut) consumption and tax revenues

Effect on	Change in price (%)	Change in consumption (%)	Change in Consumption (million packs/million kg)	Cigarette or betel nut use elasticities relative to medical costs*	Medical cost savings (%)
*Tax scenarios: Additional Health tax of NT$10*					
Cigarettes	41.47	-27.22	-609.8	0.5885	16.01
Alcohol	-	-7.50	-51.13	-	-
Betel nuts	-	-20.07	-33.13	0.0443	0.89

* All values are quoted from the estimates of Lee (2004) [47].

According to this study's estimated cigarette price elasticity of -0.6517, the NT$10 Health Tax will cause average per capita cigarette consumption to fall by 34.37 packs (approximately 27.22%), reducing national cigarette consumption by roughly 610 million packs annually and reducing medical expenditures by around 16.01%[47]. Based on a prevalence elasticity of cigarette demand of -0.15 estimated by the U.S. Centers for Disease Control and Prevention (CDC 1998) [48] and a 41.47% increase in the price of cigarettes, the NT$10 Health Tax will cause the prevalence of smoking to fall by 6%. With 3.9 million smokers in Taiwan, this implies that 234,000 people will quit smoking as the result of this tax increase. Using the estimated epidemiological analysis reported by the World Bank [49], approximately 58,500 lives will be saved due to its implementation.

In sum, a cigarette tax increase in Taiwan has the potential to substantially reduce the number of smokers, the amount of tobacco consumed, and the costs of smoking-related illnesses and premature death.

### Effects on alcohol and betel nut consumption

On average, Taiwanese consumed 9.303 kg of betel nuts and 42.93 liters of alcoholic beverages in 2001. The average retail price of betel nuts and alcoholic beverages at that time was NT$300 per kilogram and NT$88.58 per liter respectively. Employing an estimated cigarette price elasticity relative to betel nuts of -0.484 and a cigarette price elasticity relative to alcoholic beverages of -0.1881, this study calculates that the NT$10 health tax on cigarettes will cause per capita betel nut and alcoholic beverage consumption to fall by 20.07% and 7.5% respectively, reducing total annual betel nut and alcoholic beverage consumption by 33.13 million kilograms and 51.13 million liters. We estimate that this reduction in betel nut consumption will lessen medical expenditures by roughly 0.89% (see Table 2).

## Discussion

This study finds that imposition of the NT$10 Health Tax on cigarettes in 2006 will reduce cigarette consumption in Taiwan by a very significant 27.22%. This figure approximates statistics from the National Treasury, which reported in 2006 that cigarette sales had fallen by 28.63% [27].

The study also finds that a complementary relationship does exist among the three stimulants. Thus, in addition to reducing cigarette consumption, the NT$10 health tax on cigarettes will also reduce betel nut and alcoholic beverage consumption as well as the incidence of disease caused by these products. Conversely, increasing taxes on alcoholic beverages and levying a health tax on betel nuts would help to reduce cigarette consumption while also lowering alcohol and betel nut consumption.

When surtaxes have been imposed on the three addictive products, a synergistic effect will arise since each tax will actually reduce consumption of all three products. Due to the complementary relationship between smoking and betel nut use, betel nut control policy should be linked with tobacco control policy when increasing cigarette taxes. Doing so will help to curb the current rapid growth in betel nut consumption.

We conclude from this that the government should continue using cigarette taxes as a tool with which to reduce cigarette consumption. The health tax on cigarettes will not only substantially reduce cigarette consumption, but it will also make a fiscal contribution to the government. For example, it will greatly lessen the health hazards associated with such stimulants and ultimately help to achieve the goal of disease prevention. It will also encourage young smokers and smokers who are not heavily addicted to curtail their cigarette consumption, which is a primary goal of the government's tobacco control policy. Higher cigarette prices will hopefully dissuade young people from acquiring a smoking habit in their youth. (To achieve even greater success in the campaign against smoking, the government should also ensure that anti-smoking education is provided in the schools.)

### Recommendations

According to the results of this study, an NT$10 health tax on each pack of cigarettes will cause average per capita cigarette consumption to fall by approximately 34.37 packs annually, reduce Taiwan's cigarette consumption to 1.703 billion packs (calculated on the basis of the population over 15 years of age in 2005), boost government cigarette tax revenue to NT$37.118 billion, and increase the health tax on cigarettes to NT$17.03 billion. Ten percent of the increase in health tax revenue (NT$1.703 billion) could be used for smoking control, public health, and social welfare, while the remaining 90% (NT$15.327 billion) could be used to make up the current health insurance deficit. A health tax on cigarettes thus promotes social fairness and alleviates the regressive nature of cigarette taxes. As long as cigarette prices increase faster than cigarette consumption falls, the government's revenue from cigarette taxes will rise, thereby helping to fill the health insurance system's current financial shortfall. In sum, the use of health taxes on cigarettes as an anti-smoking policy tool is indeed effective and persuasive.

### The need for a health tax on alcoholic beverages and betel nuts

To date Taiwan has yet to levy any health taxes on betel nuts and alcoholic beverages. Were it to do so, the government could achieve much greater health and economic gains due to the close inherent price elasticities of cigarettes, betel nuts and alcoholic beverages and the complementary relationship between them. Therefore, we recommend the following:

Relevant laws should be amended so as to require wholesalers to collect a health tax on betel nuts. This strategy would have three advantages. First, the relatively low number of wholesalers will make it easy to keep track of and promote administrative efficiency. Second, since the wholesale level is the biggest beneficiary of the betel nut industry, taxing wholesalers will be relatively fair and free of dispute. Third, wholesalers can easily pass the cost of the health tax on to consumers, which will achieve the policy goal of reducing betel nut intake.

In the case of alcoholic beverages, a health tax should be imposed using the same approach as that adopted for cigarettes. Revenue generated from health taxes on betel nuts and alcoholic beverages could be transferred to the National Health Insurance reserve fund or used to defray the cost of betel nut and drinking control measures. Health taxes would help to compensate for the additional costs to the health insurance system caused by use of these products; they would also provide additional funding for betel nut and drinking control efforts.

## Conclusion

This study finds that cigarettes, betel nuts, and alcoholic beverages are mutually complementary; from the point of view of effects on health, these products typically exert a negative synergistic effect. Therefore, they should not be seen as separate influences. Since most betel nut users in Taiwan smoked and drank before using betel nuts, smoking and drinking can be seen as the main gateways to betel nut use.

In light of this, reducing smoking and drinking rates is the first step to lowering betel nut consumption. Conversely, reducing consumption of alcohol and cigarettes is an effective strategy with which to reduce betel nut consumption. Since the unhealthy behaviors of smoking, chewing betel nuts, and drinking typically occur together, public health authorities should develop effective health promotion programs that reflect this linkage and thereby significantly improve citizen health.

## Competing interests

The author(s) declare that they have no competing interests.

## Authors' contributions

JM. Lee is the sole author.

## Pre-publication history

The pre-publication history for this paper can be accessed here:


